# Konzeptabhängige und -unabhängige Versorgungseffekte standortspezifischer palliativer Versorgungskonzepte am Beispiel „Schmerz“

**DOI:** 10.1007/s00482-023-00754-1

**Published:** 2023-09-29

**Authors:** Sarah Peuten, Birgit Jaspers, Irmtraud Hainsch-Müller, Christoph Aulmann, Werner Schneider, Lukas Radbruch, Gülay Ateş

**Affiliations:** 1 Institut für Sozialwissenschaften, Universität Augsburg, Universitätsstr. 10, 86159 Augsburg, Deutschland; 2 Klinik für Palliativmedizin, Georg-August-Universität Göttingen, Robert-Koch-Str. 40, 37075 Göttingen, Deutschland; 3 Interdisziplinäres Zentrum für Palliativmedizin, Universitätsklinikum Augsburg, Stenglinstr. 2, 86156 Augsburg, Deutschland; 4 Klinik für Palliativmedizin, Universitätsklinikum Bonn, Venusberg-Campus 1, 53127 Bonn, Deutschland; 5 Institut für Digitale Allgemeinmedizin, Universitätsklinikum Rheinisch-Westfälische Technische Hochschule Aachen, Bahnhofstr. 14, 52064 Aachen, Deutschland

**Keywords:** Palliativversorgung, Integrierte Versorgungsstrukturen, Kooperative Versorgungsstrukturen, Versorgungskontinuität, Schnittstellenübergreifende Versorgung, Palliative care, Integrated care structures, Cooperative care structures, Continuity of care, Cross-sectoral care

## Abstract

**Hintergrund:**

An zwei Standorten wurden die palliativen Versorgungsstrukturen, sektorenübergreifenden Übergänge und Verlaufswege von Patienten mit einem palliativen Versorgungsbedarf untersucht. Der systematische Vergleich von Gemeinsamkeiten und Unterschieden anhand der exemplarischen Fokussierung auf den Themenkomplex „Schmerz“ soll Auskunft darüber geben, inwiefern diese mit standortspezifischen Palliativversorgungskonzepten (integriert und kooperativ) zusammenhängen.

**Methodik:**

Die Studie verfolgt ein Mixed-methods-Design. Neben einer Dokumentenanalyse von anonymisierten Patientenakten (*n* = 774) wurden Experteninterviews (*n* = 20), Interviews mit Patienten und Angehörigen (*n* = 60) sowie Fokusgruppen (*n* = 12) durchgeführt.

**Ergebnisse:**

Die systematische vergleichende Analyse liefert Hinweise auf konzeptunabhängige Gemeinsamkeiten (z. B. soziodemografische Verteilungen, erschwerte medikamentöse Schmerzbehandlung aufgrund rechtlicher Rahmenbedingungen) wie auch konzeptabhängige Unterschiede (z. B. Verlaufswege, erleichterte kontinuierliche Symptomkontrolle durch integrierte Versorgungsstrukturen) im Rahmen integrierter oder kooperativer Palliativversorgung.

**Diskussion:**

Gemeinsamkeiten und Unterschiede hinsichtlich der hier fokussierten Schmerzthematik bzw. der im Raum stehenden Symptomlast und ihre organisatorische Bearbeitung werden als Effekte der jeweiligen Organisationsstruktur (= konzeptabgängig) sowie konzeptunabhängiger äußerer Einflussfaktoren greifbar.

## Abstract (en)

**Background:**

Structures of palliative care, cross-sectoral transitions and care pathways of patients with palliative care needs were investigated at two sites. The systematic comparison of similarities and differences using the topic of ‘pain’ as an example is intended to provide information on the extent to which these are related to site-specific palliative care concepts (integrated and cooperative).

**Methods:**

The study follows a mixed-methods design. In addition to a document analysis of anonymised patient records (*n* = 774), expert interviews (*n* = 20), as well as interviews with patients, relatives (*n* = 60) and focus groups (*n* = 12), were conducted.

**Results:**

The systematic comparative analysis provides evidence for concept-independent commonalities (e.g. sociodemographic distribution, aggravated pain treatment) as well as concept-dependent differences (e.g. care pathways, facilitated continuous symptom control through integrated care structures) in the context of integrated or cooperative palliative care.

**Discussion:**

Commonalities and differences with regard to the topic of pain, as focused on here, and its organisational management become tangible as effects of the respective organisational structure (= concept-dependent) as well as concept-independent external influencing factors.

## Hintergrund und Fragestellung

Schmerz ist weitaus mehr als ein rein körperliches Phänomen und in der jeweils konkreten Interaktion, Kommunikation und Situation ganz unterschiedlich zu adressieren, damit dieser nachhaltig gelindert werden kann. Studien verweisen auf subjektive, kulturelle und sozial-biografische Aspekte von Schmerzerfahrung, Schmerzwahrnehmung, Schmerzdeutung, Schmerzbeschreibung sowie Schmerzverhalten bzw. Umgang mit Schmerzen [4, 5, 8, 11, 12]. So verweisen physiologische Indikatoren zwar auf subjektives Schmerzerleben, korrelieren aber häufig nicht direkt mit objektiv messbaren Parametern [14]. Schmerz ist als komplexes Leiderlebnis mit verschiedenen Dimensionen zu betrachten [9]. Regelmäßige Erfassungen von physischen, psychischen, sozialen, spirituellen Symptomen bzw. Problemen – wann immer möglich in Form von Selbsteinschätzung durch den Patienten [10, 16] – und weiteren versorgungsrelevanten Faktoren gehören zum Standard der Palliativmedizin. Patientenorientierte Bedarfe zu erfüllen und das Sicherheitsempfinden beim Wechsel zwischen verschiedenen Versorgungssettings zu stärken, stehen im Fokus von umfassenden multidisziplinären palliativen (Schmerz‑)Behandlungskonzepten [1, 6, 7, 15]. Gut organisierte Übergänge beim Wechsel vom stationären in den ambulanten Bereich durch vorausschauende Entlassplanung tragen zur Behandlungskontinuität bei. Sie haben positive Auswirkungen auf das physische sowie psychische Wohlbefinden, die Mobilität und aktive soziale Teilhabe von Patienten und deren pflegenden Angehörigen sowie deren Möglichkeiten zur Alltagsbewältigung [2, 17]. Zudem kann ein gutes Entlassmanagement unnötigen weiteren Krankenhausaufnahmen vorbeugen.

Das vom Bundesministerium für Bildung und Forschung (BMBF) geförderte Projekt „Übergänge in der Palliativversorgung – Vergleich von schnittstellenübergreifenden Versorgungsverläufen in zwei verschiedenen Krankenhauskontexten“ (TransPaC) untersuchte an zwei Standorten, Augsburg und Bonn, die Auswirkungen zweier unterschiedlicher palliativer Versorgungskonzepte auf sektorenübergreifende Übergänge aus der Perspektive des Gesundheitspersonals sowie der Patienten und pflegenden Angehörigen. Ausgehend von Unterschieden hinsichtlich des Themas Schmerz bei Aufnahme auf die Palliativstation (s. Ergebnisteil „Dokumentenanalyse“) erörtern wir anhand der erhobenen quantitativen und qualitativen Daten standortbezogene palliative Versorgungsstrukturen und Verlaufswege von Patienten mit einem palliativen Versorgungsbedarf sowie Gemeinsamkeiten und Unterschiede bei der Thematisierung von Schmerz. Schließlich untersuchen wir, wie diese mit standortspezifischen Palliativversorgungskonzepten zusammenhängen. Der Themenkomplex Schmerz bzw. Symptomlast stellt für den vorliegenden Beitrag insofern eine exemplarische Fokussierung dar, als damit nicht Fragen nach adäquater Schmerzerfassung oder -behandlung verfolgt werden sollen, sondern im Vergleich von Organisationsstrukturen über die Praxis der Versorgungsorganisation Auskunft gegeben werden kann.

## Studiendesign und Untersuchungsmethoden

Die komparativen quantitativen und qualitativen Analysen der beiden Standorte Bonn und Augsburg basieren auf systematischen Auswertungen der Thematik „Schmerz“.

## Quantitative Analysen

In eine retrospektive Auswertung von Patientenakten in Bonn und Augsburg wurden alle Patienten des Zeitraums 01.01.2016 bis 31.12.2016 eingeschlossen. Die anonymisierte Grundgesamtheit beträgt für Augsburg *n* = 356 und für Bonn *n* = 418. Aus den Krankenhausinformationssystemen wurden insbesondere die im Zuge der „Hospiz- und Palliativ-Erhebung“ (HOPE; [13]) eingegebenen Daten in eine SPSS-Datenmatrix übertragen. Neben soziodemografischen Variablen wurden krankheits- und versorgungsrelevante Aspekte harmonisiert erfasst. Die deskriptiven Tabellen beziehen sich auf gültige Antworten. Bei geringer Zellbesetzung wurden Kategorien zusammengefasst; ausgewiesen in der jeweiligen Legende der Tabellen. Somit wurden für die jeweils relevanten Variablen unter Berücksichtigung der Datenlage die jeweils entsprechenden statistischen Kennzahlen und statistischen Testverfahren für Gruppenunterschiede (Mann-Whitney-U-Test) berechnet. Die quantitativen Daten sind erste Eckdaten zur Beschreibung der Studienorte Augsburg und Bonn.

## Qualitative Interviews

Die qualitative Datengrundlage besteht aus Experteninterviews mit Versorgungsanbietenden, Patienten- und Angehörigeninterviews sowie abschließenden Fokusgruppengesprächen. Alle Interviews wurden wörtlich transkribiert. Die Datenanalyse erfolgte nach dem Ansatz der „Grounded Theory“ in MAXQDA.

### Experteninterviews

An den beiden Standorten Bonn und Augsburg wurden je zehn leitfadengestützte Interviews mit Mitarbeitenden an den Schnittstellen der palliativen Versorgung und hospizlichen Begleitung geführt – Pflegekräfte, Fachärzte im Krankenhaus, niedergelassene Hausärzte, Mitarbeitende der sozialen Dienste, der ambulanten Hospizdienste und stationären Hospize sowie der (spezialisierten) ambulanten palliativen Dienste (*n* = 20; 03/2018–07/2018). Die an institutionelle Gegebenheiten angepassten Gesprächsleitfäden umfassten Themenschwerpunkte wie institutionelle Selbstverständnisse und Routinen, organisatorische und administrative Prozesse sowie Faktoren, die Versorgungskontinuität beim Wechsel zwischen den verschiedenen Sektoren der ambulanten und stationären Versorgung erleichtern oder erschweren können.

### Interviews mit Patienten und Angehörigen

Patienten mit Anbindung an eine palliativmedizinische Versorgung oder hospizliche Begleitung im ambulanten und stationären Bereich wurden mittels eines gemeinsam entwickelten semistrukturierten Leitfadens interviewt (*n* = 60; 10/2018–06/2019). Kriterien für ein Interview waren: Patienten ohne kognitive Beeinträchtigungen, eine kürzlich erfolgte Aufnahme auf eine Palliativstation bzw. bis zu 5 Tage nach Krankenhausentlassung mit Anbindung an einen Dienst der spezialisierten ambulanten Palliativversorgung (SAPV) und/oder Anbindung an einen ambulanten Hospizdienst (AHD). Wenn Angehörige vorhanden waren, wurden diese je nach Wunsch gemeinsam mit den jeweiligen Patienten oder getrennt interviewt. Im Fokus der insgesamt 60 Gespräche stand die Zufriedenheit bzw. Unzufriedenheit beim Wechsel von einem Versorgungsbereich in den anderen. Die Kategorienbildung erfolgte induktiv aus dem Textmaterial heraus. Ziel der Interviews war es, typische Herausforderungen, Wünsche und Bedarfe der Befragten mit Blick auf bisherige Erfahrungen bei Übergängen zu ermitteln.

### Fokusgruppen

Nach Analyse der qualitativen Interviews wurden in Bonn und Augsburg jeweils sechs problemzentrierte Fokusgruppengespräche in unterschiedlichen Zusammensetzungen – hinsichtlich der verschiedenen ambulant-stationär tätigen Berufsgruppen – konzipiert und durchgeführt (*n* = 12 FG; 09/2019–07/2021). Während die ersten vier mittels problemzentrierter Fallvignetten individuelle oder systematische Herangehensweisen, Lösungskompetenzen und die weiterhin verbleibenden ungelösten Problemlagen thematisierten, lag der Fokus der letzten beiden Fokusgruppengespräche auf der Diskussion kontextsensitiver praxisrelevanter Lösungsansätze.

## Ergebnisse

Die Untersuchung konzeptabhängiger und -unabhängiger Versorgungseffekte am Beispiel „Schmerz“ findet in drei Schritten statt. Im ersten Teil der Ergebnisse werden die unterschiedlichen Voraussetzungen einer Palliativversorgung in Augsburg und Bonn dargestellt, gefolgt von einer quantitativen Dokumentenanalyse beider Palliativstationen. Diese bilden eine erste Verortungsbasis für den qualitativen Auswertungsfokus – die Erörterung und Analyse von strukturbedingten Versorgungslogiken sowie äußerer Einflussfaktoren auf die Schmerzversorgung an den beiden Standorten Augsburg und Bonn.

## Regionale Versorgungsangebote und -konzepte

### Bonn: integriertes Palliativversorgungskonzept

In Bonn wurde die Erhebung auf zwei Palliativstationen durchgeführt. Ein *integriertes Palliativversorgungskonzept* mit unterschiedlichen Angeboten der stationären und ambulanten Hospiz- und Palliativversorgung inklusive einer Zusammenarbeit mit externen regionalen und lokalen Diensten wird umgesetzt. Die Teams der ambulanten und stationären Angebote arbeiten eng zusammen; gemeinsame Büroräume auf einer Etage und teilweise personelle Doppelbesetzungen erleichtern die Kommunikation. Die Mitarbeitenden der ambulanten palliativen Dienstangebote haben aufgrund der räumlichen Nähe auch die Möglichkeit, Patienten und Angehörige vor einer Entlassung im Krankenhaus kennenzulernen, vor Ort eine Vertrauensbasis zu schaffen und gemeinsam vorausschauend zu planen. Kooperationen und ein enger Austausch bestehen mit drei ambulanten Pflegediensten (bei einem ambulanten Pflegedienst auch in Personalunion). Weitere Kooperationen gibt es mit drei stationären Pflegeeinrichtungen, einem Hospiz und einer Einrichtung für betreutes Wohnen.

### Augsburg: kooperatives Palliativversorgungskonzept

In Augsburg verfügt das Interdisziplinäre Zentrum für Palliative Versorgung am Universitätsklinikum über 18 Betten an zwei Standorten. Während die erste Palliativstation 2009 mit 10 Betten eröffnet wurde, stehen seit 2018 am Universitätsklinikum Süd weitere acht Betten zur Verfügung. Die retrospektive Analyse von Patientenakten fand somit vor dem Bestehen der zweiten Palliativstation und damit ausschließlich auf der erstgenannten Station statt. Die Zusammenarbeit zwischen den ambulanten und stationären Einrichtungen und Diensten (Tab. 1) erfolgt in Augsburg im Rahmen eines *kooperativen Palliativversorgungskonzepts* – die verschiedenen Anbieter wie SAPV oder AHD sind räumlich und örtlich getrennt, Kommunikation findet überwiegend telefonisch oder schriftlich statt. Seit einigen Jahren gibt es allerdings verstärkt Bestrebungen, die Hospiz- und Palliativarbeit in der Region noch stärker zu vernetzen und erweiterte Versorgungsangebote zu schaffen [3].

**Tab. 1:** Regionale Hospiz- und Palliativversorgungsangebote in Augsburg und Bonn. Stand 07/2020

	Augsburg	Bonn
*Stationäre Angebote in der Region*		
Palliativstationen (inkl. Umkreis von 15 km)	2	5
Hospize (inkl. Umkreis von 30 km)	1	4
*Ambulante Angebote in der Region (inkl. Umkreis von 25* *km)*		
Ambulanter Hospizdienst	10	17
Spezialisierte ambulante Palliativversorgung	2	2
Pflegedienst mit „Palliative Care Tour“	4	4

Tab. 1 gibt einen Einblick in die Versorgungsangebote am jeweiligen Standort.

## Dokumentenanalyse

Im Jahr 2016 wurden in Augsburg 356 und in Bonn 418 Patienten auf den Palliativstationen behandelt. Die soziodemografischen Verteilungen (Alter und Geschlecht) variieren nur minimal. An beiden Standorten lebte ein Großteil der Patienten nicht allein (mit Partner, Kind/Kindern etc.). Große Unterschiede zeigten sich bei den Aufnahmen auf die Palliativstationen. Krankenhausinterne Verlegungen machten in Augsburg 81 % und in Bonn 51 % der Aufnahmen aus; aus dem ambulanten Bereich kamen in Augsburg 15 %, in Bonn 39 %. In Augsburg erfolgte bei 8 % der Patienten die Aufnahme über die Notaufnahme – z. T. vorab besprochen im Rahmen eines geplanten „Umwegs“ (bspw. bei fehlenden Bettenkapazitäten, nachts oder am Wochenende). Dieser Aufnahmeweg auf die Palliativstation kommt in Bonn nicht vor und ist somit ein weiterer systematischer Unterschied zwischen den beiden Standorten. Patienten in Augsburg durchlaufen somit indirektere Wege bis zur Aufnahme auf die Palliativstation und haben ein höheres Risiko, nicht (beizeiten) als Palliativpatienten „erkannt“ und entsprechend versorgt zu werden (siehe dazu auch die Schmerzintensitäten und ECOG-Werte bei Aufnahme auf die Palliativstation in Tab. 3). Patienten in Bonn hingegen kommen wesentlich häufiger direkt und dementsprechend mit bereits im ambulanten Setting bzw. über die Schnittstelle ambulant-stationär hinweg eindeutig definiertem palliativem Versorgungsbedarf auf die Palliativstation. Die Differenz vom Zeitpunkt der Krankenhausaufnahme bis zur Verlegung auf die Palliativstation war bei internen Verlegungen hingegen gleich lang. Auch die mittlere Verweildauer auf der Palliativstation und die Gesamtaufenthaltsdauer im Krankenhaus unterscheiden sich kaum. An beiden Standorten starben ca. 60 % der Patienten auf der Palliativstation und ca. 10 % wurden in ein stationäres Hospiz entlassen. In Augsburg gingen 18 % der Patienten nach Hause und 9 % in eine Pflegeeinrichtung. In Bonn wurden 27 % nach Hause und 4 % in eine Pflegeeinrichtung entlassen (Tab. 2).

**Tab. 2:** Strukturdaten der retrospektiven Patientenakten (Auszug); TransPaC, 2016

		Augsburg	Bonn
Geschlecht	Weiblich	51 %	49 %
	Männlich	49 %	51 %
	*N*	356	418
Alter bei Aufnahme auf Station in Jahren	MW, SD, Min., Max.	70±13, 20/96	72±14, 28/102
	*N*	356	418
Wohnsituation in %	Alleinlebend	20 %	31 %
	Mit Angehörigen	65 %	60 %
	Pflegeeinrichtung etc.	11 %	7 %
	Sonstiges	3 %	2 %
	*N*	353	412
Aufnahmen von … in %	Zu Hause, Pflegeeinrichtung etc.	15 %	39 %
	Krankenhausinterne Verlegung	81 %^a^	51 %
	Externes Krankenhaus	5 %	10 %
	*N*	350	418
Patient wohnhaft in Region mit schwachen oder starken hospizlichen und palliativen Versorgungsstrukturen	Strukturschwach	19 %	14 %
	Strukturstark	81 %	86 %
	*N*	355	416
Differenz zwischen Aufnahme im Krankenhaus und Verlegung auf Palliativstation in Tagen^b^	Median, Min., Max.	10, 0/79	9, 0/83
Aufenthaltsdauer auf der Palliativstation in Tagen	Median, Min., Max.	7, 0/28	8, 0/64
Gesamtaufenthaltsdauer im Krankenhaus in Tagen	Median, Min., Max.	15, 0/87	14, 0/92
Verstorben/entlassen nach … in %	Verstorben auf Station	62 %	59 %
	Nach Hause (mit/ohne ambulante Dienstanbindung)	18 %	27 %
	Stationäres Hospiz	10 %	11 %
	Ins Pflegeheim etc.	9 %	4 %
	*N*	355	417

^a^Inkl. Notfallaufnahme, ^b^zur Berechnung des Durchschnittswerts exklusive ambulanter Aufnahmen

**Tab. 3:** Krankheitsdaten der retrospektiven Patientenakten (Auszug); TransPaC, 2016

	Augsburg	Bonn
*Grunderkrankung in %*		
Krebs	73 %	80 %
Herz-Kreislauf-System (z. B. Herzinsuffizienz)	7 %	5 %
Atmungssystem (z. B. COPD)	5 %	7 %
Nervensystem (z. B. ALS)	2 %	2 %
Verdauungssystem	3 %	2 %
Sonstiges	11 %	5 %
*N*	356	418
*ECOG in %*		
ECOG 0 und 1: normale Aktivität oder gehfähig und leichte Aktivität^a^	1 %	12 %
ECOG 2: Selbstversorgung, kann > 50 % der Wachzeit aufstehen	5 %	3 %
ECOG 3: begrenzte Selbstversorgung, > 50 % der Wachzeit bettlägerig	40 %	29 %
ECOG 4: pflegebedürftig, permanent bettlägerig	54 %	56 %
*N*	339	321
*HOPE: Schmerzerfassung bei Aufnahme*^b^		
Kein	17 %	33 %
Leicht	22 %	28 %
Mittel	36 %	23 %
Stark	25 %	16 %

^a^Aufgrund geringer Fallzahlen Zusammenlegung der Kategorien 0 und 1. ^b^Nichtparametrische Testverfahren, Mann-Whitney-U-Test aufgrund schiefer Verteilung der 4er-Schmerzskala: 1 – kein, 2 – leicht, 3 – mittel, 4 – stark.

*ECOE* Eastern Cooperative Oncology Group

Der Großteil der Patienten hatte eine Krebserkrankung. In Augsburg gab es kaum Patienten mit minimalen Einschränkungen im Alltag; in Bonn lag dieser Anteil bei 12 % (ECOG 0 und 1; Tab. 3; [5]). Die Schmerzintensität bei Aufnahme auf die Palliativstation (palliativmedizinisches Basisassessment HOPE; [13]) zeigt Unterschiede zwischen den beiden Standorten, χ2 (df3, *N* = 708) = 37,828, *p* = 0,000. Der Eta-Wert (0,219) verweist auf einen mittleren Zusammenhang zwischen Erhebungsstandort und dokumentiertem Schmerz. Die mittlere erfasste Schmerzintensität war in Bonn (M = 2,69; SD = 1,026) niedriger als in Augsburg (M = 2,22; SD = 1,076). Der Mann-Whitney-U-Test ergab einen signifikanten Unterschied der Schmerzintensität bei Aufnahme zwischen den Gruppen Augsburg und Bonn (z = 5,85, *p* = 0,000).

## Qualitative Interviews

Die systematische vergleichende Analyse liefert zum Thema Schmerz Hinweise auf konzeptunabhängige Gemeinsamkeiten wie auch konzeptabhängige Unterschiede im Rahmen integrierter oder kooperativer Palliativversorgung. Auf diesen ersten quantitativen Ergebnissen aufbauend wurde das gesamte qualitative Datenmaterial nach „Schmerz“ durchsucht, um strukturelle Gemeinsamkeiten und Unterschiede sowie äußere Einflussfaktoren bei der Thematisierung von Schmerz vertiefend zu analysieren und verstehend zu deuten.

## Konzeptunabhängige Gemeinsamkeiten

### Vorher-Nachher-Kontrastierung gelingender bzw. nicht gelingender Symptomkontrolle

Sprechen Patienten und Angehörige nach Aufnahme auf die Palliativstation über Schmerzen, stehen neben konkreten Schmerzschilderungen globale Schmerzerzählungen im Vordergrund. Mittels kontrastierender bzw. vergleichender Vorher-Nachher-Schilderungen veranschaulichen die Befragten an beiden Standorten, wie es vor dem Versorgungswechsel auf die Palliativstation war und welche Veränderungen mit diesem einhergehen. Der Wechsel ermöglicht die Einnahme einer reflektierend-vergleichenden Perspektive auf und Kommunikation über Schmerzlasten.

Folgende Zitate verdeutlichen eindrücklich den positiven Effekt gelingender (palliativmedizinischer) Schmerzbehandlung mit Blick auf die Rückgewinnung von Autonomie und Alltagskompetenzen sowie das subjektive Sicherheitsempfinden:

„Das lange Leben ist nicht das Thema. *Das Thema ist Leben, aber ohne Schmerz, ohne, ohne, ohne, ohne, ohne, ohne, ohne Einschränkung.*“ (…) Ich habe nichts mehr gegessen. Ich wurde immer magerer. Das ist auch vorbei. Ich kann jetzt wieder essen. Ich habe Appetit. (…) Der Schmerz hat die Lebensgeister zerstört, also kaputt gemacht. (…) die Lebensqualität ist erheblich verbessert. (…) dass der Schmerz genommen wurde und offenbar gezielt der Schmerz behandelt wird. (…) nach zwei Tagen waren die Schmerzen weg und da war ich glücklich. *Ich war zum ersten Mal glücklich im Krankenhaus *(lacht). Es ist skurril, aber es ist so. (Patient, Palliativstation Bonn, Tumorerkrankung)

„Da kann ich ein Wort dazu sagen. *Die Sicherheit. Ja die Sicherheit, in guten Händen zu sein*, ist eigentlich das Ausschlaggebende gewesen. (…) Hier ist man aufgehoben. *Ja, auf einer anderen Station, würde ich sagen, ist man zwar auch unter Kontrolle, aber nicht in dem Fall so aufgehoben*.“ (Patientin, Palliativstation Augsburg, Tumorerkrankung)

Kritik an unzureichender Schmerzmedikation erfolgt in Bonn und Augsburg eng verknüpft mit vielschichtigen, nicht primär auf das konkrete Schmerzerleben bezogenen Negativerfahrungen. Unzulängliche, unpersönliche Kommunikation ist hierbei ebenso von Bedeutung wie nicht ernst genommen bzw. vertröstet zu werden oder an Entscheidungen nicht angemessen beteiligt zu sein:

„*Der Austausch, das Zuhören*, dass der Arzt dem Patienten zuhört, dass die Schwester dem Patienten zuhört. Dass, ja, einfachste Dinge, die auf Station [Station Nr.] überhaupt nicht funktionieren (…). *Wo nicht geguckt wird, wie der Schmerz ist, *wo Tabletten, die kurzzeitig, nur zwei Stunden schmerzlindernd wirken, (…) wird gesagt: ‚Das wirkt sechs Stunden und Sie kriegen bloß drei Stück am Tag. *Da müssen Sie jetzt zurechtkommen damit und den Rest müssen Sie aushalten.*‘ Und so was geht nicht.“ (Patientin, Palliativstation Augsburg, Tumorerkrankung)

Auch seitens der Versorgenden sind mit Blick auf Übergänge Kontrastierungen im Sprechen über Schmerz zu beobachten. Eigene professionelle Selbstverständnisse sowie Arbeitsweisen sind in der Gegenüberstellung von einem „Wir-Hier“ (z. B. auf der Palliativstation) und „Die-Dort“ (z. B. auf den Normalstationen) von großer Bedeutung:

„Die Dame haben wir am Donnerstag kennengelernt, die kam mit Schmerzen. (…) Dann kommen wir heute auf Station, weil wir gucken wollten, wie es ihr geht. ‚Ja, sie muss heute wieder heim.‘ *Das ist halt von Station jetzt so gewesen. Das war jetzt nicht so, wie ich es mir gewünscht hätte*, aber die Station hat halt gemeint, für sie ist alles erledigt.“ (Experteninterview, palliativmedizinischer Dienst Augsburg)

## Erschwerte medikamentöse Schmerzbehandlung durch rechtliche Rahmenbedingungen und BtM-Verordnungspraxis

Nicht neu, aber aufgrund neuer rechtlicher Rahmenbedingungen führen Krankenhausentlassungen aufgrund des Verbots der Mitgabe von Betäubungsmitteln (BtM) zur akuten Unterversorgung von „Schmerz“ im häuslichen Bereich. In den teilnehmenden Einrichtungen in Bonn und Augsburg verordnen nur Chef- oder Oberärzte BtM-Rezepte. In Bonn schildern Sozialdienstmitarbeitende eindringlich ihren hohen Kommunikationsaufwand bzgl. BtM-Rezepten:

„*Da wurden die Opiatmedikationen noch mal umdosiert am Tag der Entlassung* (…), *damit das dann plötzlich kein Opiat mehr war. Ohne Bedarfstropfen. *Mit dem Kommentar von einer Oberärztin dieser Klinik an den Patienten: ‚Das bekommen sie zu Hause nirgendwo verschrieben. (…) Deswegen machen wir das so.‘ Wir hatten in der [Stationsname] einen Oberarzt, mit dem wir ständig zusammenarbeiten, der hat uns über seine Assistenzärzte ausrichten lassen, er würde keine BtM-Rezepte mehr ausstellen.“ (Fokusgruppe 3, krankenhausinterne Sozialdienste und Palliativdienste, Bonn)

## Schnittstellenbedingte Einflussfaktoren auf medikamentöse Schmerzbehandlung und Versorgungskontinuität

Im ambulanten Bereich listen an beiden Standorten Experten die „klassischen Schnittstellenproblematiken“ wie Wochenendentlassungen, fehlende oder fehlerhaft ausgefüllte Dokumente und Verordnungen, schwer erreichbare Ansprechpartner, erschwerte (Weiter‑)Versorgung von Patienten mit komplexen Wunden, Tracheostoma, Port oder Dekubitus, keine oder zu geringe Mitgabe von Medikamenten und/oder häufig fehlende Bedarfsmedikation auf. Patienten in Bonn und Augsburg berichten weiterhin von Ängsten und Sorgen zu potenziellen Problemen im häuslichen Umfeld (Schmerzkontrolle, Hilfsmittel oder Ansprechpartner). Dies ist nicht der Fall, wenn ein ambulanter Hospiz- oder spezialisierter Palliativdienst eingebunden ist. Lösungsansätze bzw. Ideen zur Verbesserung beziehen sich beispielsweise auf teilstationäre Angebote, Brückenpflege, die Klärung des Vorgehens bei weiterer Verschlechterung/Eskalation vor Entlassung, kontinuierliche Ansprechpersonen sowie den Ausbau palliativmedizinischer Fortbildungen (auch mit Fokus auf nichtonkologische Erkrankungen).

## Konzeptabhängige Unterschiede

### Erschwerte Symptomkontrolle durch zu späte Palliativanbindung

In Augsburg wird im Rahmen der Experteninterviews kritisiert, dass eine adäquate kontinuierliche Symptomkontrolle oftmals nicht früh genug ermöglicht wird, da Patienten erst bei hoher Symptomlast, in sehr reduziertem Allgemeinzustand oder mit zeitlicher Verzögerung palliativmedizinisch angebunden bzw. weiterversorgt werden. Das betrifft sowohl den ambulanten wie auch den stationären Bereich:

„Na, viele verspätete. *Viele verspätete. *Sieht man ja auch an den Verweildauern im Haus, wie lange die teilweise schon stationär sind, dann haben wir sie einen Tag und dann sterben sie. Dann sehe ich ja schon, was verkehrt gelaufen ist, im Endeffekt, ja. *Also ich finde, wir müssten früher angebunden werden*.“ (Experteninterview, Palliativstation Augsburg)

„Dass ein Krankenhaus entlässt *ohne Schmerzmedikation* und das nicht kommuniziert *und wir zwei Tage später bestellt sind*. Das sind ganz komische Sachen, die da entstehen können. Und wo einfach *jemand zu Hause ist und viel Stress und viel Not hat*.“ (Experteninterview Augsburg, ambulant)

Diese Problematik wird in Bonn so nicht benannt und verweist auf Zusammenhänge zwischen unterschiedlichen Versorgungsstrukturen und Aufnahme‑/Anbindungswegen, -zeitpunkten und palliativen Versorgungsverläufen. Integrierte Versorgungsstrukturen ermöglichen nicht nur das frühzeitige Kennenlernen von Patienten seitens der ambulanten Dienstleistungserbringer vor Entlassung und damit eine möglichst passgenaue Entlassplanung bzw. Weiterversorgung, sondern auch die direkte Aufnahme bereits bekannter Patienten, dann, wenn eine adäquate Symptomkontrolle im häuslichen Umfeld nicht (mehr) gewährleistet ist.

## Erleichterte kontinuierliche Symptomkontrolle durch integrierte Versorgungsstrukturen

In Bonn stützen die Patienten- und Experteninterviews, dass eine gelingende Symptomkontrolle im Sinne umfassender Linderung von Leid bei intersektoralen Übergängen v. a. dann fließend verlaufen kann, wenn stabile, integrierte regionale Versorgungsstrukturen bestehen. Diese reduzieren das Risiko von Versorgungsbrüchen und erleichtern auch die gezielte Anbindung nicht integrierter bzw. kooperierender ambulanter Akteure:

„Und wir dann so *quasi nahtlos *von hier [dem Krankenhaus, Anm. d. A.] dann *die Weiterversorgung in den eigenen SAPV Team halt gewährleisten können*. Dann auch *die Weiterverordnung der Medikation damit einigermaßen im Griff haben*. Und es dann halt mehr oder weniger darauf ankommt, den Hausarzt darauf vorzubereiten, dass eben noch ein neuer Dienst dann halt noch mit am Patienten dran ist. Wo die allermeisten da eher sehr froh drüber sind.“ (Experteninterview, Bonn, stationäre Einrichtung)

Schnittstellenübergreifende Zusammenarbeit in Augsburg beruht hingegen weniger auf Vertrauen in personenunabhängige, verlässliche Strukturen, sondern vielmehr auf Vertrauen in Personen (das dann wegfällt bzw. unsicher wird, wenn Personen wechseln) – darauf, dass man sich kennt und weiß, was mit wem wann in welcher Form (nicht) zu bewerkstelligen ist:

„Meine Erfahrungen: *Habe ich ein Gesicht, kenne ich jemanden persönlich, und habe ich meinen gleichen Ansprechpartner, läuft es gut. (…) Es hat auch einfach ‚was mit Vertrauen zu tun‘*. Und auch mit auch mal was möglich machen, von beiden Seiten jetzt. Also es läuft vieles auf der persönlichen Ebene.“ (Experteninterview Augsburg, ambulant)

## Diskussion

Im Rahmen der quantitativen und qualitativen Datenanalyse werden Gemeinsamkeiten und Unterschiede hinsichtlich der hier fokussierten Schmerzthematik bzw. der im Raum stehenden Symptomlast und ihre organisatorische Bearbeitung als Effekte der jeweiligen Organisationsstruktur (= konzeptabgängig) sowie konzeptunabhängiger äußerer Einflussfaktoren (rechtliche Rahmenbedingungen) greifbar. In Bonn befördern integrierte Versorgungsstrukturen kurzwegige, verlässliche sektorenübergreifende Zusammenarbeit und begünstigen eine kontinuierliche Schmerz- bzw. Symptombehandlung auch maßgeblich deshalb, da Interventionen im ambulanten Bereich Teil des organisationalen Handlungsfelds sind und der Nachvollzug von Versorgungsverläufen somit über stationäre Grenzen hinaus möglich wird. In Augsburg sind gelingende Übergänge durch mehr Unsicherheitsfaktoren gekennzeichnet. Kooperative Versorgungsstrukturen bedingen eher personenabhängige, sektorenbezogene Zusammenarbeit und erschweren eine möglichst frühe und kontinuierliche palliativmedizinische Versorgung. Das zeigt sich auch an den unterschiedlichen Aufnahme- und Verlaufswegen (s. „Dokumentenanalyse“) und der in Augsburg vonseiten der Versorgenden beklagten „späten“ palliativen Anbindung von Patienten mit hoher Symptomlast (s. „Konzeptabhängige Unterschiede“). Integrierte palliative Versorgungsstrukturen hingegen sind nicht nur weniger anfällig für Versorgungsbrüche, sie stärken sektorenübergreifend Palliativwissen und begünstigen eine frühzeitige routinierte palliative Versorgung („early integration“). Ambulante und stationäre Übergänge gelingen umso besser, je höher der Anteil hospizlicher und palliativer Versorgungsangebote sowie externer Kooperationspartnerschaften ist. Jedoch tragen die aktuellen BtM-Rezeptverordnungen bzw. der Umgang damit an beiden Standorten zu akuter Unterversorgung bei. Ungelöst bleibt dieses Problem insbesondere für nicht-angebundene Patienten mit einem palliativmedizinischen Versorgungsbedarf, die weiterhin einen Großteil ausmachen.

Bei der Dateninterpretation ist limitierend zu berücksichtigen, dass in Bonn die Symptomerfassung bei Aufnahme auf die Palliativstation durch Ärzte erfolgt, und in Augsburg durch Pflegekräfte. Im Rahmen der Studie konnte nicht untersucht werden, ob und inwiefern möglicherweise unterschiedliche Anforderungen an die Symptomdokumentation und damit zusammenhängend je spezifisches Palliativwissen hinsichtlich der Bedeutung und Relevanz von Schmerz zu den signifikanten Unterschieden bei Schmerzintensitäten beitragen.

## Fazit für die Praxis

- Palliativmedizinische Anbindungen mit 24/7-Rufbereitschaft beugen einer akuten Unterversorgung mit Schmerzmedikation vor und können einerseits eine Notfallaufnahme kurz nach Krankenhausentlassung verhindern sowie eine direkte Aufnahme auf eine Palliativstation begünstigen.
- Der Vergleich zweier unterschiedlicher Palliativversorgungskonzepte – integriert und kooperativ – untermauert den Effekt von Organisationsstrukturen für eine kontinuierliche schnittstellenübergreifende Versorgung.
- Externe Faktoren, wie etwa gesetzliche Rahmenbedingungen oder fehlende Versorgungsangebote, haben unabhängig vom jeweiligen palliativen Versorgungskonzept und der jeweiligen Organisationsstruktur unmittelbar Einfluss auf Versorgungskontinuität.

## References

[1] Adl S, Weltermann BM, Kuching A, Martin C, Korbonits G, Hopp HW (2001) Difficulties in the transfer of drug therapy from inpatient to ambulatory treatment. Gesundheitswesen 63:597–601 11607867 10.1055/s-2001-17874

[2] Ateş G, Ebenau AF, Busa C, Csikos Á, Hasselaar J, Jaspers B, Menten J, Payne S, Van Beek K, Varey S, Groot M, Radbruch L (2018) “Never at ease”—family carers within integrated palliative care: a multinational, mixed method study. BMC Palliat Care 17:39 29490657 10.1186/s12904-018-0291-7 PMC5831577

[3] https://www.ahpv.de/. Zugegriffen: 24. Mai 2022

[4] Dreßke S (2021) Empfindliche Körper. Kopfschmerzpraktiken zwischen Alltag und Medizin. Transkript, Bielefeld

[5] Dreßke S, Göckenjahn G (2009) Schmerz und Rollenübernahme: Überlegungen zur Funktion und Deutung von Schmerzen in der medizinischen Rehabilitation und der Sterbendenversorgung. Psychol Gesellschaftskritik 33:91–109

[6] Glare PA, Auret KA, Aggarwal G, Clark KJ, Pickstock SE, Lickiss JN (2003) The interface between palliative medicine and specialists in acute-care hospitals: boundaries, bridges and challenges. Med J Aust 179:S29–31 12964933 10.5694/j.1326-5377.2003.tb05574.x

[7] Husain A, Barbera L, Howell D (2013) Advanced lung cancer patients’ experience with continuity of care and supportive care needs. Support Care Cancer 21:1351–1358 23274923 10.1007/s00520-012-1673-7

[8] Le Breton D (2003) Schmerz. Eine Kulturgeschichte. Diaphanes, Zürich/Berlin

[9] Maio G, Bozzaro C, Eichinger T (Hrsg) (2015) Leid und Schmerz. Konzeptionelle Annäherungen und medizinische Implikationen. Verlag Karl Alber, München

[10] Murtagh FE, Ramsenthaler C, Firth A, Groeneveld EI, Lovell N, Simon ST et al (2019) A brief, patient- and proxy-reported outcome measure in advanced illness: validity, reliability and responsiveness of the integrated palliative care outcome scale (IPOS). Palliat Med 33:1045–1057 31185804 10.1177/0269216319854264 PMC6691591

[11] Müller-Busch HC (2015) Schmerz und Leid in der Palliativmedizin. In: Maio G, Bozzaro C, Eichinger T (Hrsg) Leid und Schmerz. Konzeptionelle Annäherungen und medizinische Implikationen. Verlag Karl Alber, München, S 288–311

[12] Oechsle K, Goerth K, Bokemeyer C, Mehnert A (2013) Symptom burden in palliative care patients: perspectives of patients, their family caregivers, and their attending physicians. Support Care Cancer 21:1955–1962 23430011 10.1007/s00520-013-1747-1

[13] Radbruch L, Nauck F, Ostgathe C, Lindena G (2009) HOPE Handbuch zu Dokumentation und Qualitätsmanagement in der Hospiz- und Palliativversorgung. Der Hospiz Verlag, Wuppertal

[14] Simon ST, Altfelder N, Alt-Epping B, Bausewein C, Weingärtner V, Voltz R, Ostgathe C, Radbruch L, Lindena G, Nauck F (2014) Is breathlessness what the professional says it is? Analysis of patient and professionals’ assessments from a German nationwide register. Support Care Cancer 22:1825–1832 24535239 10.1007/s00520-014-2131-5

[15] Soelver L, Rydahl-Hansen S, Oestergaard B (2014) Identifying factors significant to continuity in basic palliative hospital care—from the perspective of patients with advanced cancer. J Psychosoc Oncol 32:167–188 24364876 10.1080/07347332.2013.873999

[16] Stiel S, Matthes ME, Bertram L, Ostgathe C, Elsner F, Radbruch L (2010) Validierung der neuen Fassung des Minimalen Dokumentationssystems (MIDOS²) für Patienten in der Palliativmedizin: Deutsche Version der Edmonton Symptom Assessment Scale (ESAS). Schmerz 24:596–604 20882300 10.1007/s00482-010-0972-5

[17] Wells N, Pasero C, McCaffery M (2008) Improving the quality of care through pain assessment and management. In: Hughes RG (Hrsg) Patient safety and quality: an evidence-based handbook for nurses. Agency for Healthcare Research and Quality, Rockville (MD) 21328759

